# The National Medical Union Dinner

**Published:** 1914-07-04

**Authors:** 


### THE NATIONAL MEDICAL UNION DINNER.

On the evening of Saturday of last week members of the National Medical Union with their friends dined together at the Monico Restaurant, Piccadilly Circus, W. Dr. N. T. Greenyer, F.R.C.S. (Brighton), who had been chairman of the first general meeting of the Union earlier in the day, presided. Dr. Playfair, proposing "The National Medical Union," said the National Medical Union had been called into being by the National Insurance Act. Manchester had led the way in the attempt to bind together those who were opposed to the medical provisions of that Act; in 1912 there was formed in that city a body of medical men, the chief item of its policy being no service of any kind under the National Insurance Act. The same idea was in the forefront of the policy of the National Medical Union. Manchester was to be congratulated on its foresight in recognising that a union of non-panel medical men was needed; he believed that as time went on it would be felt more and more that such a union was necessary, not only for the protection of its members, but also for upholding all that was best and worthiest in the medical profession.

Edinburgh, he was glad to be able to state, had closely followed the lead of Manchester. On January 6, 1913, at a meeting of all the medical practitioners in Edinburgh a resolution not to accept service under the Act was carried by a majority. Sad to say, a panel was nevertheless formed. But the very next night a meeting of medical men opposed to the Act was held in the Royal College of Physicians, and the Edinburgh Medical Union was formed. In June of last year the members of this union had felt that the time had come for a union of all non-panel men; negotiations were accordingly extended to Manchester and London, and the National Medical Union was established. That body was now regularly constituted and had a definite course of action; what was wanted now was to strengthen and widen its operations by inducing non-panel men to join without further delay. He had been surprised while sitting at the Union's general meeting to learn that in England there were 12,976 men not on the panels. On the panels there were 11,820. In Scotland there were 2,151 not on the panels, as against 1,756 who were on. The grand total for England, Scotland, and Wales was 15,663 non-panel and 14,472 on the panels. Out of so many non-panel men there ought to be three or four thousand willing to join the union and pay a subscription. Such a number would be sufficient to place it on a sound financial basis. It was very difficult to arouse the medical profession. Its members in general were not good business men, and had not yet seen the necessity of combination in order to get what they were justly entitled to, and to defend the high ideals which to some of them were more than mere financial success. What was needed was one or two enthusiasts to go through the length and breadth of the land. He coupled with the toast the names of Dr. Porter, Dr. Brassey Brierley, and their chairman that evening, Dr. Greenyer.

Dr. Porter, in responding to the toast, said that instinct had led medical men before the beginning of 1913 to stand up and renounce the National Insurance Act. Reason came into play afterwards, but unfortunately was not sufficient to prevent a certain number falling. In the National Medical Union they knew they were fighting for the highest ideals of their profession. In Edinburgh they had done a large amount of spade work, and this was having its effect upon the mind of the public.

Mr. Brassey Brierley said that the honour and dignity of the noble profession to which they belonged were threatened, and the threat was a serious one, for it was backed up by commercial interests. When they had been told in Manchester officially that resistance to the Act was useless they had determined to have a new organisation to fight the battle of the non-panel men. At three days' notice they had held a meeting with a thousand people present. Seven hundred men joined the new association from that one meeting. But then came the great betrayal, a huge blunder made by self-seeking men who put financial considerations before everything else. For a time after that the non-panel men had lain low, until Dr. Carswell had visited Manchester, and then the National Medical Union had been established there as a permanent institution. That union had already enormous power, and the time was near at hand when the Government—and he did not care whether it was the present Government or a changed one—would have to come to it for advice and help in any reconstruction of the present Act or drafting of a new one for national medical insurance. Let it go ahead, secure an addition to its membership, and it would be a power in the country that would never be resisted either by Parliament or public.

The chairman said that the National Medical Union recognised that there was, and always would be, a section of the community for whom an efficient medical service was essential. But the master mind by which such a national service could be obtained had not been found at Westminster. Still, those present had hope that such a mind was in existence, and that when the time was ripe a complete scheme satisfactory to all would be established. It seemed strange that hitherto no attention had been given to the vast machinery already working at the highest level for the sick poor. In his opinion that material must be incorporated in any national service if satisfaction was to be given to the profession and public.

The other principal toast of the evening was that of "The Guests." Dr. Greenyer, who proposed it, coupled the toast with the names of Dr. F. J. Smith and Dr. Oliver.

Dr. F. J. Smith, in responding to the toast, said he was perfectly certain that the panel men were looking almost entirely to the £ s. d. aspect of their profession. One effect of the Act was that if he, as a teacher, wanted a student to learn something for the sake of his profession, the student would say, not to the teacher but to his companions directly the teacher's back was turned, "Profession be hanged! I can earn £1,000 a year by having 3,000 patients on my panel." Consequently, the effect of the Act was a tendency not to help the education of students and bring a better class of students into the profession, but just the reverse.

What had astonished him and made him at times almost ashamed to think that he belonged to the College of Physicians was their utter apathy. It was only after a great deal of trouble that he had succeeded in persuading the College to appoint a committee to consult with the College of Surgeons and go into the matter. But if the Colleges did not bestir themselves he felt certain they would get a class of medical students who would learn as little as they possibly could. They learned little enough now, but they would learn still less then, and would say to anyone who tried to impress upon them the necessity of learning that little well: "Why should I? I do not want it. I can earn a good living without." Already a very large number of panel men did not do their work properly—nor, indeed, do the work they were paid to do. The Union had been born nearly two years ago; he looked upon that day's proceedings as its rebirth. If it had a good solid programme, and insisted on its men doing their work decently and in order, it was quite possible that in future developments it might be appealed to as to the way a Bill should be drafted. That would be a very proud position for the Union to occupy. He hoped it would come. His own personal opinion was that the income limit was the first thing that required revision. If it were brought down to £100 he thought something might be done. All must recognise that those with incomes of £100 per annum or less were not in a position to pay for reasonable medical advice without combination. These must combine and insure so that the healthy could help the sick to pay their proper fees.

